# Harnessing synthetic biology to expand chemical diversity of antibiotics

**DOI:** 10.1093/synbio/ysaa029

**Published:** 2021-01-05

**Authors:** Fatima Enam

**Affiliations:** Department of Microbiology & Immunology, Stanford University School of Medicine, Stanford, CA 94305, USA

Antibiotic resistance is one of the greatest challenges facing public health.^1^ Historically, glycopeptide antibiotics (GPA) have been used in the treatment of infections caused by Gram-positive pathogens, including multiresistant *Staphylococcus aureus* (MRSA) infections and enterococcal infections, which are resistant to beta-lactams and other antibiotics. Rising GPA resistance has led to the discovery and clinical development of synthetic second-generation glycopeptides but more needs to be done to stand up against the evolution of resistance mechanisms. In a recent publication in *Nature Communications*, a team of scientists led by Gerard Wright at McMaster University in Canada, developed a clever synthetic biology platform for the production and discovery of novel GPAs.^2^

GPAs are heptapeptides that are naturally synthesized in actinobacteria, orchestrated by co-localized genes in the genome, called biosynthetic gene clusters (BGCs), and a host of other genes for modification, regulation and transport. Although next-generation sequencing technologies and machine-learning tools have enabled the identification of a wealth of BGCs in bacterial genomes, low or no yields of GPAs from parental actinomycetes, lack of heterologous hosts for expressing BGCs and difficulty in cloning these large constructs (>70 kb) has kept these GPAs elusive.

Xu *et al.* engineered *Streptomyces coelicolor*, a genetically tractable microorganism, that contains the biosynthetic machinery and precursors required for GPA biosynthesis, including pathways for nonproteinogenic amino acid components to create the GPAHex chassis. To clone the large BGCs and increase selection efficiency, they developed an optimized transformation associated recombination (TAR) system that allows isolation and manipulation of large DNA constructs.^3^

The TAR system relies on a copy number control replicon that can be conditionally induced for high copy numbers. The system utilizes a *ura3* counter-selection marker, which was also optimized by introducing additional transcriptional initiation sites between the TATA box and the start codon to maximize *ura3* transcription.

To demonstrate the synthesis of a GPA, they targeted corbomycin, a Type V GPA. Corbomycin was discovered by phylogeny guided genome mining, but further development was hindered by low titers in the producer Streptomyces strain. Xu *et al.* cloned a 76 kb region, constituting the peptide scaffold and six TISs, into the *S. coelicolor* chassis strain via *E. coli*-yeast triparental mating. Growth inhibition was observed against *Bacillus subtilis* and the production titer was 65.4 mg/l, 19-fold higher compared to the parental Streptomyces strain.

The authors also used the GPAHex platform for the discovery of a GPA in an Amycolatopsis strain that shares homology with the teicoplanin class of GPAs but is transcriptionally inactive in the parental strain. They cloned the scaffold including four TISs into the chassis strain to express GP1416 that displayed antibacterial activity against *B. subtilis*. Chromatographic analysis revealed that the structure of this novel GPA was indeed analogous to teicoplanin, with additional modification of specific sites. They took this discovery platform a step further by identifying a cryptic Type V GPA produced by a number of Streptomyces strains and successfully expressing the GPA with good yields. The structure revealed an aliphatic valine residue which is uncommon in other Type V GPAs, composed entirely of aromatic residues.

This GPAHex system expands the repertoire of GPAs and provides a platform for the discovery and development of GPAs and other peptide natural products, especially from organisms that lack genetic tractability. The application of their optimized TAR cloning system is broad-reaching in that can be used for assembly of large constructs with higher efficiencies in many synthetic biology applications and functional/structural genomics studies.

*Conflict of interest statement*. None declared.
